# Gut dysbiosis in severe mental illness and chronic fatigue: a novel trans-diagnostic construct? A systematic review and meta-analysis

**DOI:** 10.1038/s41380-021-01032-1

**Published:** 2021-02-08

**Authors:** Jenelle Marcelle Safadi, Alice M. G. Quinton, Belinda R. Lennox, Philip W. J. Burnet, Amedeo Minichino

**Affiliations:** 1 Cornell University, Ithaca, NY USA; 2 Department of Psychiatry, University of Oxford, Oxford, UK

**Keywords:** Diagnostic markers, Psychiatric disorders

## Abstract

Reduced gut-microbial diversity (“gut dysbiosis”) has been associated with an anhedonic/amotivational syndrome (“sickness behavior”) that manifests across severe mental disorders and represent the key clinical feature of chronic fatigue. In this systematic review and meta-analysis, we investigated differences in proxy biomarkers of gut dysbiosis in patients with severe mental illness and chronic fatigue vs. controls and the association of these biomarkers with sickness behavior across diagnostic categories. Following PRISMA guidelines, we searched from inception to April 2020 for all the studies investigating proxy biomarkers of gut dysbiosis in patients with severe mental illness and chronic fatigue. Data were independently extracted by multiple observers, and a random-mixed model was used for the analysis. Heterogeneity was assessed with the *I*^2^ index. Thirty-three studies were included in the systematic review; nineteen in the meta-analysis (*N* = 2758 patients and *N* = 1847 healthy controls). When compared to controls, patients showed increased levels of zonulin (four studies reporting data on bipolar disorder and depression, SMD = 0.97; 95% Cl = 0.10–1.85; *P* = 0.03, *I*^2^ = 86.61%), lipopolysaccharide (two studies reporting data on chronic fatigue and depression, SMD = 0.77; 95% Cl = 0.42–1.12; *P* < 0.01; *I*^2^ = 0%), antibodies against endotoxin (seven studies reporting data on bipolar disorder, depression, schizophrenia, and chronic fatigue, SMD = 0.99; 95% CI = 0.27–1.70; *P* < 0.01, *I*^2^ = 97.14%), sCD14 (six studies reporting data on bipolar disorder, depression, schizophrenia, and chronic fatigue, SMD = 0.54; 95% Cl 0.16–0.81; *P* < 0.01, *I*^2^ = 90.68%), LBP (LBP, two studies reporting data on chronic fatigue and depression, SMD = 0.87; 95% Cl = 0.25–1.48; *P* < 0.01*; I*^2^ = 56.80%), alpha-1-antitripsin (six studies reporting data on bipolar disorder, depression, and schizophrenia, SMD = 1.23; 95% Cl = 0.57–1.88; *P* < 0.01, *I*^2^: 89.25%). Elevated levels of gut dysbiosis markers positively correlated with severity of sickness behavior in patients with severe mental illness and chronic fatigue. Our findings suggest that gut dysbiosis may underlie symptoms of sickness behavior across traditional diagnostic boundaries. Future investigations should validate these findings comparing the performances of the trans-diagnostic vs. categorical approach. This will facilitate treatment breakthrough in an area of unmet clinical need.

## Introduction

In the past two decades, the view of severe mental illnesses as brain-centered disorders has been successfully challenged [1]. A growing body of evidence shows that a number of peripheral influences are at play, suggesting that a whole-body perspective might offer greater insight into the understanding of mental health conditions [2, 3].

The gut–brain axis, with the relatively recent characterization of the human gut microbiome, is emerging as a key path on the bidirectional communication network between peripheral and central physiological functions [4]. Understanding the relevance of gut microbiome modifications for clinical features of severe mental illness is a research priority, given its modifiable nature and the possibility of unveiling new therapeutic targets in areas of unmet need.

**Fig. 1:**
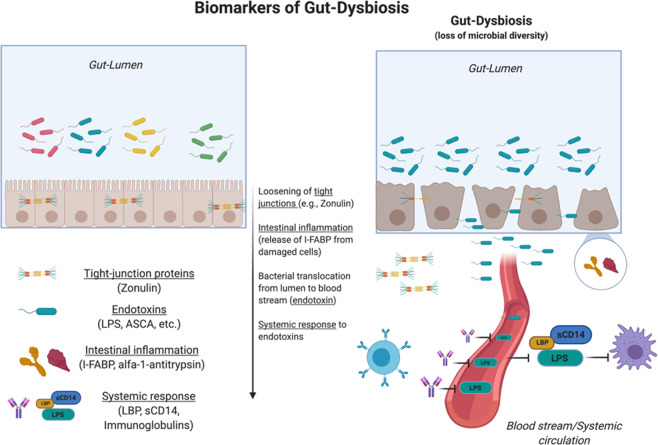
Proxy biomarkers of gut dysbiosis. Gut dysbiosis (i.e., reduced gut-microbial diversity) has been shown to trigger: **a** a local inflammatory response (alpha-1-antitrypsin; I-FABP); **b** loosening of tight-junction proteins (zonulin); **c** translocation of bacterial endotoxin from the gut lumen to the bloodstream (LPS, ASCA, etc.); **d** activation of a systemic low-grade inflammation (LBP, sCD14, antibodies against bacterial endotoxin). Experimental models of gut dysbiosis showed a causative link with symptoms of sickness behavior (more details in main text).

Reduced gut-microbial diversity (“gut dysbiosis”) has been associated with a number of detrimental health outcomes, including severe mental illness and chronic fatigue [4]. This association appears to occur, at least in part, via a biological pathway that includes the activation of a peripheral pro-inflammatory response [5–7]. Animal models showed that gut dysbiosis triggers a chronic low-grade pro-inflammatory status in the host by increasing the permeability of the gut barrier (“leaky gut”) and by facilitating the translocation of bacterial antigens into the bloodstream (“endotoxemia”) (Fig. 1) [5–7]. In both animals and experimental models in otherwise healthy subjects, endotoxemia manifests with a range of flu-like symptoms (fatigue, anhedonia, loss of motivation), which most authors refer to as “sickness behavior” [8, 9].

Diagnostic terminology aside [10], symptoms of sickness behavior can be found in all major mental illnesses and beyond, such as in chronic fatigue (Table 1). A recent review [11] nicely summarizes findings from a number of neuroimaging and electrophysiological studies, highlighting a shared central pathophysiology for key sickness behavior symptoms, such as anhedonia and avolition, across different diagnostic categories. Peripheral mechanisms are also shared, with meta-analytic evidence showing increased pro-inflammatory cytokines levels in schizophrenia and depression [12], and bipolar disorder [13], and an association with severity of sickness behavior symptoms [14]. Similar findings were reported for chronic fatigue [15].

**Table 1:** Symptoms of sickness behavior in schizophrenia (negative symptoms), depression, bipolar disorder (depressive symptoms), and chronic fatigue.

	Sickness behavior	Schizophrenia (negative symptoms)	Depression and bipolar disorder (depressive symptoms)	Chronic fatigue syndrome
Fatigue	X	X	X	X
Malaise	X		X	X
Depressed mood	X		X	
Sleep disturbance: insomnia and/or hypersomnia	X		X	X
Impaired concentration	X		X	X
Curbing of interests	X	X	X	X
Diminished social drive	X	X	X	X
Diminished emotional range		X		
Anhedonia	X		X	
Psychomotor retardation or agitation	X	X	X	
Anorexia	X		X	X
Hyperalgesia	X			X
Pyrexia (fever)	X			X Swollen throat and/or lymph nodes
Scaled used for assessment		PANSS negative, SANS	HAMD, BPRS, BDI, CDSS	Fibrofatigue Scale

*FibroFatigue Scale* Fibromyalgia and Chronic Fatigue Syndrome Rating Scale, *HAM-D* Hamilton Depression Rating Scale, *MADRS* Montgomery–Åsberg Depression Rating Scale, *PANSS-NSS* Positive and Negative Syndrome Scale (negative symptom subscale).

The existence of a shared central and peripheral pathophysiology across major psychiatric illnesses is not surprising, considering the common genetic vulnerability [16]. This common ground may translate in clinical features that manifest across diagnostic boundaries, as hypothesized by a number of clinical and scientific high-profile initiative, such as the Research Domain Criteria [17].

Based on aforementioned considerations, we hypothesize that gut dysbiosis might underlie symptoms of sickness behavior in severe mental illness and beyond, such as in chronic fatigue.

To provide ground for this hypothesis, we conducted a systematic review and meta-analysis aimed at: (i) summarizing the evidence on differences in proxy markers of gut dysbiosis (Fig. 1) in severe mental illnesses (schizophrenia, depression, and bipolar disorder) and chronic fatigue vs. controls; and (ii) investigating the association between these peripheral biomarkers and the severity of sickness behavior symptoms across diagnostic boundaries.

## Methods

This systematic review and meta-analysis was conducted in accordance with the PRISMA guidelines (PRISMA Flowchart in Supplementary). Protocol was registered in PROSPERO. The search for published studies was conducted from inception to April 2020, in Web of Science and PubMed.

Titles and abstracts were imported to Mendeley. We included for full-text analysis: (i) case–control studies reporting data on proxy markers of gut dysbiosis in patients vs. controls; and (ii) studies investigating the association of these biomarkers with symptom severity and treatment response. We included studies investigating patients with a DSM or ICD-codified diagnosis of schizophrenia, depression, and bipolar disorder. We also included studies investigating chronic fatigue syndrome as defined by internationally validated criteria, such as the Fukuda diagnostic criteria. All these conditions manifest with symptoms of sickness behavior, as outlined in Table 1.

The full list of biomarkers was a-priori agreed with an expert (PWJB) after a preliminary search of the literature, and included: tight-junction proteins (zonulin, claudin, etc.); endotoxins (lipopolysaccharide (LPS)); proteins related to the immune response to bacterial antigens (lipopolysaccharide binding protein (LBP) and sCD14); antibodies against bacterial endotoxin, such as anti-Saccharomyces cerevisiae antibodies (Ig-ASCA); proteins related to intestinal inflammation, such as alpha-1-antitrypsin (A-1-AT) and intestinal fatty-acid binding protein (I-FABP).

No restrictions were set in terms of age, duration of illness, and medication status. Reasons for exclusion are documented in the Supplementary. Authors were contacted as needed to determine inclusion. The entire search process was conducted independently by JMS and AMGQ; any disagreements were resolved by AM. Data extraction was independently conducted by JMS and AMGQ.

### Outcomes and meta-analyses

Our primary outcome was to compare differences in circulating levels of proxy markers of gut dysbiosis in patients vs. healthy controls. Our secondary outcomes were: (i) to report data on the relationship between proxy markers of gut dysbiosis and the severity of sickness behavior symptoms (measured across different diagnostic categories, see Table 1); (ii) to report data on the association between modifications of proxy markers of gut dysbiosis and response to treatment. A quantitative synthesis of the differences in proxy markers of gut dysbiosis between patients and controls was provided when data from two or more studies were available for a specific biomarker. Medians, standard errors, and interquartile ranges were transformed to means and SDs, following a validated procedure [18]. When necessary, data were extracted from graphs using a web-based tool (WebPlot Digitizer). All meta-analyses were conducted in R, and the standardized mean difference (SMD) was used as the summary statistic. Heterogeneity between studies was estimated using Higgin’s *I*^2^. Sensitivity analyses were performed by sequentially removing single studies and re-running the analysis. When possible, we explored the effect of medication status on meta-analytic findings by comparing differences in medicated and unmedicated patients. Finally, we reran all the analyses by: (1) removing studies investigating chronic fatigue, to explore if findings were specific for severe mental illness only; (2) removing studies that explicitly included participants in the hypomanic or manic phase of bipolar illness, which do not manifest symptoms of sickness behavior.

## Results

**Fig. 2:**
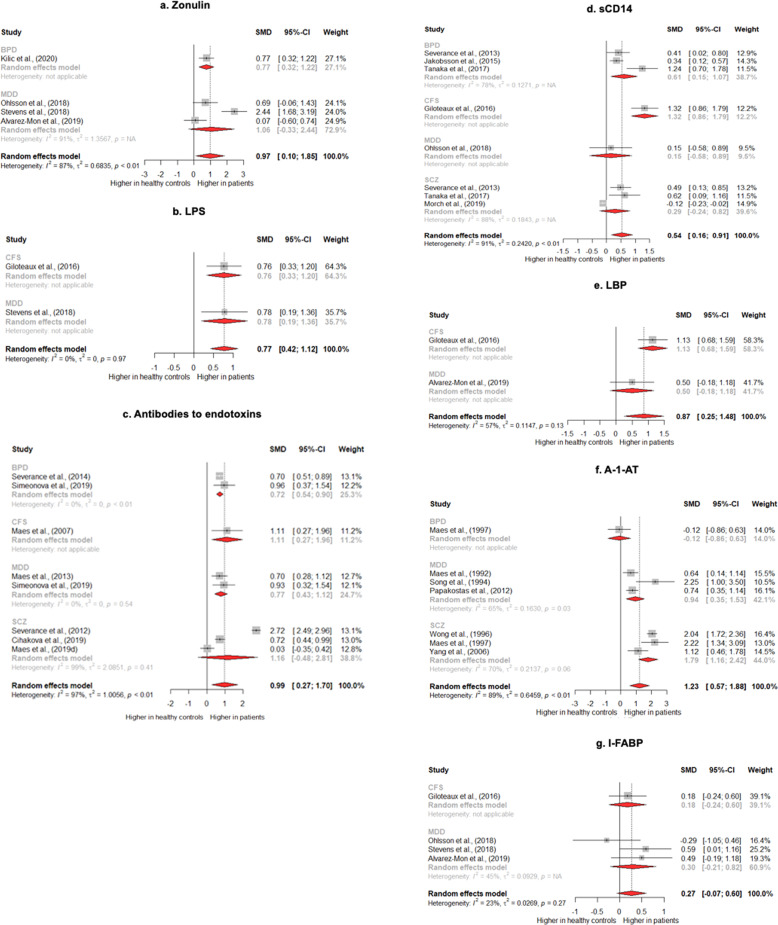
Forest plots of proxy biomarker of gut dysbiosis in severe mental illness and chronic fatigue. **a** Levels of circulating zonulin in patients with CFS and MDD vs. controls; **b** levels of circulating LPS in patients with CFS and MDD vs. controls; **c** levels of circulating antibodies to endotoxins in patient with BPD, CFS, MDD; SCZ vs. controls; **d** levels of circulating sCD14 in patients with BPD, CFS, MDD, SCZ vs. controls; **e** Levels of circulating LBP in patients with CFS and MDD vs. controls; **f** levels of circulating A-1-AT in patients with BPD, MDD, SCZ vs. controls; **g** levels of circulating I-FABP in patients with CFS and MDD vs. controls. A-1-AT alpha-1-antitrypsin, BPD bipolar disorder, CFS chronic fatigue syndrome, I-FABP intestinal fatty-acid binding protein, LBP lipopolysaccharide binding protein, LPS lipopolysaccharide, MDD major depressive disorder, sCD14 soluble CD14, SCZ schizophrenia.

Thirty-three studies met the inclusion criteria [9, 13, 19–49]. Of these, 19 studies [9, 13, 19–22, 27, 29, 32–36, 38, 39, 41, 42, 46, 49] provided data and were included in the meta-analysis (*N* = 2758 patients and *N* = 1847 healthy controls). It was possible to provide a quantitative synthesis for the following biomarkers: zonulin, LPS, LBP, sCD14, antibodies against bacterial endotoxins, A-1-AT, and I-FABP (Fig. 2).

### Differences in proxy markers of gut dysbiosis in patients vs. controls

Four studies provided data on circulating levels of zonulin in patients (*N* = 98) vs. controls (*N* = 100) [33, 36, 38, 39]. Three of these studies were conducted on patients with depression [36, 38, 39] and one on bipolar disorder [33]. Across these four studies, the pooled estimate showed a significant increase in zonulin in patients vs. controls (SMD = 0.97; 95% Cl = 0.10–1.85; *P* = 0.03), with evidence of high heterogeneity (*I*^2^ = 86.61%) (Fig. 2a). Sequentially removing single studies from the analysis did not reduce heterogeneity. However, one study [36], included also patients with anxiety disorders; when this study was removed, the heterogeneity dropped to a moderate level (*I*^2^ = 32.48%), with results being still significant (SMD = 0.55; 95% Cl = −0.12–0.97; *P* = 0.011).

Only two studies provided data on circulating levels of endotoxins (LPS) in patients (*N* = 71) vs. controls (*N* = 66) [36, 46]. One of these studies was conducted on patients with depression [36] and the other on chronic fatigue [46]. The pooled estimate showed increased levels of LPS in patients vs. controls (SMD = 0.77; 95% Cl = 0.42–1.12; *P* < 0.01; *I*^2^ = 0%) (Fig. 2b). A third study [37], not included in the quantitative synthesis, measured LPS in root canal samples of patients with depression and controls. Results from this study were in line with the overall pooled estimate for LPS (Table 2), showing increased levels of LPS in patients vs. controls.

**Table 2:** Summary of findings by diagnostic category.

Study	Subjects	Gender (%F)	Markers	Stage	Phase	BMI	Smoking	Main findings
Schizophrenia								
Wong et al. [19]	SCZ (Cohort 1) = 98 SCZ (Cohort 2) = 50 HC = 91	SCZ: 0% HC: 0%	1. A-1-AT (blood)	Mixed	Mixed	Not reported	Not reported	1. ↑ A-1-AT in SCZ vs. HC
Maes et al. [20]	SCZ = 27 HC = 11		1. A-1-AT (blood)	Mixed/unclear	Mixed/unclear	Not reported	Not reported	1. =levels of A-1-AT in SCZ vs. HC
Yang et al. [21]	SCZ = 22 HC = 20	SCZ: 0% HC: 0%	1. A-1-AT (blood)	Mixed/unclear	Mixed/unclear	Not reported	Note reported	1. ↑ A-1-AT in SCZ vs. HC
Severance et al. [47]	SCZ = 363 HC = 207	SCZ: 32.7% HC: 72.9%	1. ASCA (blood)	Mixed	Mixed	Not reported	Not reported	1. ↑ ASCA in SCZ vs. HC
Severance et al. [22]	SCZ = 141 HC = 39	SCZ: 39.7% HC: 71.8%	1. sCD14 (blood) 2. LBP (blood)	Unclear	Unclear	SCZ: 30.54 ± 0.61	SCZ: 58.9% HC: 17.9%	1. ↑ sCD14 in SCZ vs. HC 2. =LBP between SCZ vs. HC
Dickerson et al. [23]	SCZ = 249 rPsych = 79 HC = 260	SCZ: 31% rPsych: 34% HC: 61%	1. ASCA (blood)	Mixed Chronic (SCZ) and recent onset of psychosis (rPsych)	Mixed	SCZ: 30.8 ± 7.5 rPsych: 26.1 ± 7.5 HC: 27.5 ± 6.8	SCZ: 64% rPsych: 41% HC: 15%	1. ↑ ASCA in SCZ vs. HC 2. ↓ ASCA in rPsych vs. HC
Dickerson et al. [24]	Patients = 210 [SCZ = 90 BPD = 72 MDD = 48] Recent suicide attempt (rSA) HC = 72	Patients: 46.6% HC: 64%	1. IgA-ASCA (blood) 2. IgA LPS (blood)	Mixed/unclear	Mixed/unclear	Patients: 31.19 ± 8.7 HC: 28.1 ± 7.3	Patients: 41.4% HC: 14%	1. ↑ ASCA IgG in patients with rSA vs. HC 2. ↑ LPS-IgA in patients with rSA vs. HC
Weber et al. [25]	SCZ = 80 HC = 80	SCZ: 15% HC: 15%	1. sCD14 (blood) 2. LBP (blood)	“Early stages”	Outpatient	N/A	N/A	1. ↑ sCD14 in SCZ vs. HC 2. =LBP in SCZ vs. HC
Delaney et al. [26]	Psychosis (Psych) = 42 Clinical high risk (CHR) = 17 HC = 33	Psych: 36% CHR: 35% HC: 57.5%	1. IgA to LPS (blood) 2. IgG to LPS (blood) 3. IgM to LPS (blood)	Mixed/unclear	Mixed/unclear	Psych: 27.8 ± 7.88 CHR: 23.87 ± 4.02 HC: 24.84 ± 6.26	N/A	1. =IgA, IgG, and IgM in Psych vs. HC 2. =IgA, IgG, and IgM in CHR vs. HC
Ciháková et al. [27]	SCZ = 160 HC = 80	SCZ: 38% HC: 36%	1. ASCA IgA (blood) 2. ASCA IgG (blood)	Mixed/unclear	Mixed/unclear	N/A	N/A	1. ↑ ASCA IgG in SCZ vs. HC
Mørch et al. [28]	SCZ = 675 HC = 647	SCZ: 39.85% HC: 46.83%	1. sCD14 (Blood)	Mixed/unclear “The patients were recruited during early phase after an acute episode (post-acute episode)”	Mixed “Mostly from outpatient clinics”	SCZ: 26.4 ± 5.2 HC: 24.7 ± 3.5	SCZ: 46% HC: 9%	1. ↓ sCD14 in SCZ vs. HC
Maes et al. [29]	SCZ = 80 [*n* = 40 deficit schizophrenia] HC = 38	Not reported	1. IgA gram-negative bacteria 2. (Blood) 3. IgM gram-negative bacteria 4. (Blood)	Mixed/unclear	Outpatients	Not reported	Not reported	1. ↑ IgA to four gram-negative bacteria in deficit SCZ vs. non-deficit SCZ and HC 2. ↑ IgM to five gram-negative bacteria in deficit SCZ vs. non-deficit SCZ and HC
Maes et al. [30]	SCZ = 78 HC = 40	PT: 46.8% HC: 75%	1. IgM zonulin 2. (Blood) 3. IgM occludin 4. (Blood) 5. IgM e-cadherin 6. (Blood) 7. IgA gram-negative bacteria 8. (Blood)	Multi-episode	Mixed/unclear	SCZ: 24.4 ± 5.14 HC: 24 ± 4.3	SCZ: 6.3% HC: 5%	1. ↑ IgM to zonulin in SCZ vs. HC 2. ↑ IgM to occludin in deficit vs. non-deficit SCZ and HC
Maes et al. [31]	SCZ = 79 HC = 40	PT: 46.8% HC: 75%	1. IgA tight junctions + adherens junctions (blood) 2. e-Cadherin, occludin, claudin-5, beta-catenin 3. (Blood) 4. IgA gram-negative (blood)	Mixed/unclear “stabilized phase of illness”	Mixed/unclear	SCZ: 41.2 ± 11.1 HC: 24.0 ± 4.3	SCZ: 6.3% HC: 5%	1. ↑ IgA to TJ/AJs in deficit SCZ vs. non-deficit SCZ and HC 2. ↑ IgA to gram-negative bacteria in deficit SCZ vs. non-deficit SCZ 3. ↑ IgA occludin associated with deficit SCZ vs. non-deficit SCZ
Bipolar disorder								
Maes et al. [20]	BPD = 23 HC = 10		1. A-1-AT (blood)	Mixed/unclear	Mixed/unclear	Not reported	Not reported	1. =levels of A-1-AT in BPD vs. HC
Severance et al. [22]	BPD = 75 HC = 39	BPD: 69.3% HC: 71.8%	1. sCD14 (blood) 2. LBP (blood)	Unclear	Unclear	BPD: 27.17 ± 0.77	BPD: 34.7% HC: 17.9%	1. ↑ sCD14 in BPD vs. HC 2. =LBP in BPD vs. HC 3. ↑ LBP in BPD vs. SCZ
Severance et al. [32]	BPD = 264 HC = 207	BPD: 69.7% HC: 72.9%	1. IgG-ASCA (blood)	Mixed/unclear	Outpatients	Not reported	Not reported	1. ASCA IgG in patients vs. controls
Jakobsson et al. [9]	BPD = 221 HC = 112	BPD: 62% HC: 55%	1. sCD14 (blood)	Mixed/unclear	Outpatients	BPD: 24.86 HC: 23.46	BPD: 33% HC: 15.2%	1. ↑ sCD14 in BD vs. HC
Tanaka et al. [13]	BPD = 32 HC = 32	BPD: 56% HC: 56%	1. sCD14 (blood)	Mixed/unclear	Outpatients	N/A	BPD: 52.5% HC: 40.6%	1. ↑ sCD14 in BPD vs. HC
Kılıç et al. [33]	BPD = 41 HC = 41	BPD: 56.1% HC: 48.7%	1. Zonulin (blood) 2. Claudin-5 (blood)	Mixed/unclear	Mixed/unclear	BPD: 26.4 ± 2.9 HC: 25.3 ± 3.3	BPD: 43.9% HC: 36.5%	1. ↑ Zonulin in BPD vs. HC 2. ↑ Claudin-5 in BPD vs. HC
Depression								
Maes et al. [34]	MDD with melancholy (M) = 22 MDD without M = 20 HC = 26	N/A but controlled for sex	1. A-1-AT (blood)	Multi-episode	Mixed/unclear	N/A	N/A	1. ↑ A-1-AT in MDD with and without M vs. HC
Maes et al. [20]	MDD = 29 HC = 21	Not reported	1. A-1-AT (blood)	Mixed/unclear	Mixed/unclear	Not reported	Not reported	1. =levels of A-1-AT in MDD vs. HC
Papakostas et al. [35]	Pilot study: MDD = 36 HC = 43 Replication study: MDD = 34	Pilot study: MDD = 36.1% HC = 67.4% Replication study: MDD = 55.8%	1. A-1-AT (blood)	Multi-episode	Mixed/unclear	Pilot: MDD = 27.7 ± 5.8 HC = 24.4 ± 3.5 Replication: MDD = 30.6 ± 9.7	N/A	1. ↑ A-1-AT in MDD patients vs. HC (both pilot and replication study)
Maes et al. [48]	MDD = 113 HC = 28	MDD: 52.2% HC: 67.9%	1. IgM to LPS (blood) 2. IgA to LPS (blood)	Mixed/unclear	Outpatients	N/A, but BMI > 30 excluded	Smokers excluded from study	1. ↑ IgM and IgA to LPS in MDD vs. HC
Stevens et al. [36]	MDD or ANX = 22 HC = 27	N/A	1. LPS (blood) 2. Zonulin (blood) 3. I-FABP (blood)	Mixed/unclear	Mixed/unclear	N/A	N/A	1. ↑ LPS in MDD/ANX vs. HC 2. ↑ Zonulin in MDD/ ANX vs. HC 3. ↑ I-FABP in MDD/ ANX vs. HC
Gomes et al. [37]	MDD = 24 HC = 23	N/A	1. LPS (root canal)	Mixed/unclear	Outpatients	N/A	N/A	1. ↑ LPS in MDD vs. HC 2. ↑ LPS associated with severity of depression
Ohlsson et al. [38]	MDD = 13 Recent suicide attempt (rSA) = 54 HC = 17	MDD: 53.8% rSA: 55.5% HC: 47.1%	1. Zonulin (blood) 2. I-FABP (blood) 3. sCD14 (blood)	Mixed/unclear	Mixed/unclear	MDD: 25.9 ± 8.7 rSA: 25.7 ± 4.4 HC: 23.1 ± 3.1	N/A	1. ↓ Zonulin in rSA vs. HC 2. ↑ I-FABP in rSA vs. HC 3. =Zonulin, I-FABP, and sCD14 in MDD vs. HC
Alvarez-Mon et al. [39]	MDD = 22 HC = 14	MDD: 59.1% HC: 57.1%	1. LBP (blood) 2. Zonulin (blood) 3. I-FABP (blood)	Mixed/unclear	1. Outpatient	MDD: 26.45 ± 4.04 HC: 25.26 ± 3.87	MDD: 22.7% HC: 21.4%	1. ↑ LBP in MDD vs. HC 2. ↑ I-FABP in MDD vs. HC 3. =Zonulin in MDD vs. HC
Maes et al. [40]	MOOD = 96 [27 BPD1, 25 BPD2, 44 MDD] HC = 22	MOOD: 46.8% HC: 36.3%	1. IgM/IgA gram-negative bacteria (blood)	Multi-episode	Outpatient	MOOD: 25.4 ± 2.8 HC: 25.3 ± 3.8	MOOD: 79.1% HC: 95.4%	1. ↑ IgM/IgA to gram-negative bacteria in MOOD patients vs. HC
Simeonova et al. [41]	MDD = 44 HC = 30	MDD: 47.7% HC: 66.6%	1. IgM/IgA to gram-negative bacteria (blood)	Multi-episode	Mixed/unclear	MDD: 24.83 ± 3.01 HC: 24.74 ± 2.72	MDD: 93% HC: 96.5%	1. ↑ IgM/IgA in MDD vs. HC
Song et al. [49]	Depressed = 10 HC = 8	Depressed: N/A HC: 50%	1. A-1-AT (blood)	Unclear	Unclear	N/A	N/A	1. ↑ A-1-AT in depressed patients vs. HC
Chronic fatigue								
Maes et al. [42]	CFS = 15 HC = 11	CFS: 66.7% HC: 72.7%	1. IgM to LPS* (blood) 2. IgA to LPS* (blood) *LPS from seven different bacterial strains	N/A	Outpatient	N/A	N/A	1. ↑ IgA LPS in all 7 bacteria in CFS vs. HC 2. ↑ IgM LPS in 3/7 bacteria in CFS vs. HC
Maes et al. [43]	CFS = 41	CFS: 82.9%	1. IgM LPS (blood) 2. IgA LPS (blood)	N/A	Outpatient	N/A	N/A	1. ↓ in IgM and IgA to LPS post-NAIOS treatment 2. ↓ in FFS symptoms post- NAIOS treatment, especially in younger patients with shorter DOI
Maes et al. [44]	CFS = 90 CF, undiagnosed = 31	CFS = 83.3% CF = 71%	1. IgA LPS (blood) 2. IgM LPS (blood)	N/A	Mixed/unclear	N/A	N/A	1. ↑ IgM and IgA LPS in CFS vs. CF
Maes et al. [45]	CFS = 90 CF, undiagnosed = 31	CFS: 80.9% CF: 73.5%	1. IgM LPS (blood) 2. IgA LPS (blood)	N/A	Mixed**/**unclear	N/A	N/A	1. ↑ levels of IgM for LPS in CFS patients vs. CF patients 2. ↑ levels of IgA for LPS in patients vs. CF patients
Giloteaux et al. [46]	CFS = 49 HC = 39	CFS: 77.6% HC: 76.9%	1. LPS (blood) 2. I-FABP (blood) 3. sCD14 (blood) 4. LBP (blood)	N/A	Mixed/unclear	CFS: 25.5 ± 4.9 HC: 27.1 ± 6.1	N/A	1. ↑ LPS in CFS vs. HC 2. = I-FABP in CFS v. HC 3. ↑ levels of sCD14 in CFS vs. HC 4. ↑ levels of LBP in CFS vs. HC

↑ = significantly higher, ↓ = significantly lower, = means no significant difference.

*A-1-AT* alpha-1-antitrypsin, *ANX* anxiety disorder, *BPD* bipolar disorder, *CF* formally undiagnosed chronic fatigue, *CFS* chronic fatigue syndrome, *HC* healthy controls, *I-FABP* intestinal fatty-acid binding protein, *LBP* lipopolysaccharide binding protein, *LPS* lipopolysaccharide, *MDD* major depressive disorder, *MOOD* mood disorders, *noSA* no suicide attempt, *PT* patients, *rPsych* recent onset of psychosis, *rSA* recent suicide attempt, *SCZ* schizophrenia.

Seven studies [27, 29, 32, 41, 42, 47, 48] provided data on circulating levels of antibodies against bacterial endotoxins in patients (*N* = 1104) vs. controls (*N* = 603). Three of these studies reported data on patients with schizophrenia [27, 31, 47], one on bipolar disorder [32], one on depression [48], one on bipolar disorder and depression [41], and one on chronic fatigue [42].

The pooled estimate showed increased levels of antibodies against bacterial endotoxins in patients vs. controls (SMD = 0.99; 95% CI = 0.27–1.70; *P* < 0.01), with evidence of high heterogeneity (*I*^2^ = 97.14%) (Fig. 2c). This high heterogeneity was explained by removing one study [47], with results remaining significant (SMD = 0.67; 95% Cl 0.44–0.89; *P* < 0.01*; I*^2^ = 55.67%). Three studies, two on chronic fatigue [42, 45] and one on a mixed sample of bipolar disorder and major depression [40], investigated levels of antibodies against bacterial endotoxins in patients vs. controls, but did not provide data. All these three studies reported increased levels of antibodies against bacterial endotoxins in patients vs. controls, in line with our pooled estimate (Table 2).

Six studies [9, 13, 22, 28, 38, 46] provided data on circulating levels of sCD14 in patients (*N* = 1234) vs. controls (*N* = 962). Two of these studies reported data on patients with bipolar disorder and schizophrenia [13, 22], one on bipolar disorder [9], one on schizophrenia [28], one on chronic fatigue [46], and one on depression [38]. The pooled estimate showed increased levels of sCD14 in patients vs. controls (SMD = 0.54; 95% Cl 0.16–0.81; *P* < 0.01), with evidence of high heterogeneity (*I*^2^ = 90.68%) (Fig. 2d). Sequentially removing single studies from the analysis did not reduce heterogeneity.

Only two studies [39, 46] provided data on circulating levels of LBP in patients (*N* = 71) vs. controls (*N* = 51). One study was conducted on patients with depression [39] and the other on chronic fatigue [46]. The pooled estimate showed increased levels of LBP in patients vs. controls (SMD = 0.87; 95% Cl = 0.25–1.48; *P* < 0.01*; I*^2^ = 56.80%) (Fig. 2e). Two additional studies that were not included in the quantitative synthesis investigated differences in LBP in patients vs. controls [22, 25]. The first study reported not significantly higher levels of LBP in bipolar disorder patients compared to controls; however, a quantitative summary was not available [22]. The other study [25] was excluded from the meta-analysis because it was conducted on patients before the onset of schizophrenia (prodrome), showing no differences in LBP circulating levels between patients and controls.

Six studies [19–21, 34, 35, 49] provided data on circulating levels of A-1-AT in patients (*N* = 342) vs. controls (*N* = 209) (Fig. 2f). One of these studies was conducted on patients with bipolar disorder and schizophrenia [20], two on schizophrenia [19, 21], and three on depression [34, 35, 49]. The pooled estimate showed increased levels of A-1-AT in patients vs. controls (SMD = 1.23; 95% Cl = 0.57–1.88; *P* < 0.01), with evidence of high heterogeneity (*I*^2^: 89.25%). Sequentially removing single studies from the analysis did not reduce heterogeneity.

Four studies [36, 38, 39, 46] provided data on circulating levels of I-FABP in patients (*N* = 101) vs. controls (*N* = 92). One of these studies [46] was conducted on patients with chronic fatigue and three on depression [36, 38, 39]. The pooled estimate showed no significant differences in I-FABP levels of patients vs. controls (SMD = 0.27; 95% Cl −0.07–0.60; *P* = 0.12; *I*^2^ = 23.05%).

In summary, our pooled estimates showed increased circulating levels of: tight-junction proteins (zonulin, four studies reporting data on bipolar disorder and depression); bacterial endotoxins (LPS, two studies reporting data on chronic fatigue and depression); intestinal inflammation markers (A-1-AT, six studies reporting data on bipolar disorder, depression, and schizophrenia); gut-related systemic inflammation markers (LBP, two studies reporting data on chronic fatigue and depression; and sCD14, six studies reporting data on bipolar disorder, depression, schizophrenia, and chronic fatigue); antibodies against endotoxins (seven studies reporting data on bipolar disorder, depression, schizophrenia, and chronic fatigue), in patients compared to controls.

Significance of pooled estimates did not change when studies on chronic fatigue and hypomanic/manic patients with bipolar illness were excluded from the quantitative analyses. Medication status did not influence findings (Supplementary material). As outlined in Table 2, BMI and smoking status were accounted for by in the majority of included studies (Supplementary material).

### Association between proxy markers of gut dysbiosis, severity of sickness behavior symptoms, and response to treatment

The association between circulating levels of proxy markers of gut dysbiosis and severity of sickness behavior symptoms was investigated by 14 studies [13, 23, 28–32, 34, 37, 38, 28–32, 40–42, 44] (Table 3). Of these studies, seven were conducted on patients with schizophrenia [13, 23, 28–31, 40], five on depression [34, 37, 38, 40, 41], four on bipolar disorder [13, 32, 40, 41], and two on chronic fatigue syndrome [42, 44].

**Table 3:** Association between proxy markers of gut dysbiosis and severity of sickness behavior in schizophrenia, bipolar disorder, major depression, and chronic fatigue.

	CFS	Schizophrenia	BD	MDD
Tight-junction proteins		+ + [30, 31]		
Circulating endotoxin				+ [37]
sCD14		= = [13, 28]	= [13]	
Antibodies against bacterial endotoxins	+ + [42, 44]	= [23] + + + [29, 31, 40]	= [41] + + [32, 40]	+ + [40, 41]
Intestinal inflammation				+ + [34, 38]

The association between levels of the biomarker and the severity of sickness behavior is (+) significantly positive or (=) not significant.

Four studies [29–31, 40] showed that greater circulating levels of proxy markers of gut dysbiosis (tight-junction proteins, endotoxins, antibodies against endotoxins) were significantly associated with more severe and persistent negative symptoms (deficit schizophrenia) among patients with schizophrenia. This association was not found in studies including patients with less severe negative symptoms [13, 23, 28].

Six studies [32, 34, 37, 38, 40, 41] out of seven [13, 32, 34, 37, 38, 40, 41] found that increased circulating levels of proxy markers of gut dysbiosis (A1-AT, I-FABP, endotoxins, antibodies against endotoxins) were significantly associated with more severe symptoms of depression in MDD [34, 37, 38, 40, 41] and bipolar disorder [32, 40].

Finally, two studies found that increased circulating levels of proxy markers of gut dysbiosis (antibodies against endotoxins) were significantly associated with more severe symptoms of CFS [42, 44].

Only one study, conducted in patients with chronic fatigue, investigated the relationship between proxy markers of gut dysbiosis and treatment response in patients. This study showed normalization of circulating levels of antibodies against endotoxin after successful treatment [43].

## Discussion

To the best of our knowledge this is the first systematic review and meta-analysis investigating proxy biomarkers of gut dysbiosis in severe mental illness and chronic fatigue.

Gut dysbiosis biomarkers were increased in patients vs. controls and associated with more severe symptoms of sickness behavior across diagnostic categories, independent of medication status.

The pooled estimates showed that patients, when compared to controls, had increased circulating levels of the tight-junction protein zonulin, the endotoxin LPS, the gut-related systemic inflammatory proteins LBP and sCD14, antibodies against endotoxins, and the acute phase protein A-1-AT.

Zonulin is a tight-junction protein and key regulator of intestinal permeability, with increased circulating levels suggesting a compromised intestinal barrier [6]. Our finding of increased levels of zonulin in patients vs. controls is in line with recent pathophysiological models of psychiatric disorders, where increased permeability of biological barriers, including the blood–brain barrier, is at play [50, 51]. According to these models, the loss of integrity of these protective layers (“leakiness”) would result in increased passage to the bloodstream and the brain of “unwanted” material, including “false” neurotransmitters, pro-inflammatory stimuli, and bacterial endotoxins (“endotoxemia”). Accordingly, circulating levels of the bacterial endotoxin LPS were increased in patients compared to controls. The peripheral increase in LPS would also explain findings of increased innate and adaptive response to circulating endotoxins (sCD14/LBP and antibodies) in patients. When the innate immune system detects endotoxins in the blood, the Toll-Like-Receptor-4 pathway is activated in monocytes and the sCD14/LBP complex is released into circulation [52]. sCD14 binds to LPS in the cell wall of gram-negative bacteria alongside its co-receptor LBP (Fig. 1) [53, 54], stimulating the release of pro-inflammatory cytokines [52]. Similarly, the adaptive immune response is triggered, resulting in increased release of antibodies against bacterial endotoxins in the bloodstream.

In experimental models of endotoxemia, the chronic pro-inflammatory status that follows bacterial translocation results in a whole-body response that manifest with sickness behavior in the attempt to conserve energy and recover. In line with these models and consistent with the literature on inflammation and psychiatric disorders, we found that increased levels of proxy biomarkers of gut dysbiosis were positively associated with severity of sickness behavior in severe mental illness and chronic fatigue.

Among these biomarkers, those related to the adaptive immune response to endotoxins were most consistently associated with sickness behavior across diagnoses (Table 3). Several epidemiological studies have shown an association between autoimmune diseases and severe mental illnesses [3, 55, 56], and an autoimmune root has been suggested for CFS. Gut dysbiosis and structurally distinct endotoxins have been shown to trigger autoimmunity [57, 58], with downstream consequences of neuroinflammation [5, 59] and clinical phenotypes of sickness behavior.

Finally, we found evidence of increased intestinal inflammation in patients compared to controls. The acute phase protein A-1-AT was increased in patients, indicating greater inflammation and protein loss (Fig. 2f) [60, 61]. In contrast, the pooled estimate for I-FABP did not reveal a significant difference between patients and controls (Fig. 2g). I-FABP is uniquely localized in the gut and released with enterocyte damage, so we expected it to be elevated with inflammation of the gastrointestinal (GI) tract [39, 62]. A-1-AT is released by different tissues in response to acute damage; one potential explanation for these findings is that A-1-AT may be elevated as a consequence of a systemic inflammation, rather than localized (GI) inflammation.

Medications showed no influence on the elevation of gut dysbiosis biomarkers (see Supplementary). This finding challenges reports that nonantibiotic drugs [36], including second generation antipsychotics [63] and SSRIs [64] alter the composition of the gut microbiome. It is possible that drug-induced changes in gut microbiome composition are not sufficient to impact the pathophysiological path characterized by compromised intestinal barrier integrity, bacterial translocations, and activation of the systemic immune response.

Altogether, our findings suggest that gut dysbiosis occurs in severe mental illness and chronic fatigue and might underlie symptoms of sickness behaviors.

Some of the investigated markers, such as antibodies against endotoxins, were altered across all the included clinical conditions. This suggests that gut dysbiosis could represent a novel trans-diagnostic marker and a potential trans-therapeutic target for symptoms of sickness behavior. Future prospective studies should validate the trans-diagnostic relevance of gut dysbiosis following the recently introduced TRANSD criteria (please see ref. [65]).

Future studies aimed at clarifying the trans-diagnostic relevance of gut dysbiosis for symptoms sickness behavior are of upmost importance as research based on traditional diagnostic categories failed to identify biological correlates and effective therapeutic targets for these highly invalidating symptoms.

### Limitations

This systematic review and meta-analysis has limitations. First, we used proxy biomarkers of gut dysbiosis rather than direct measures of reduced microbial diversity. We used this approach for two reasons: (1) larger number of evidence and greater potential for replication of results obtained on blood-based biomarkers; (2) issues in gut microbiome datasets of individual data quality and inherent heterogeneity of individual datasets.

The use of proxy biomarkers comes with the limitation that some mediators, albeit commonly used in the scientific literature on the topic, might suffer from complex and hardly accountable confounding factors. This is the case of zonulin and sCD14, which alterations can be triggered by mechanisms other than gut dysbiosis. However, among the included biomarkers, LPS, LBP, and antibodies to endotoxin, which are more strictly related to gut dysbiosis, were consistently increased in patients vs. controls, therefore accounting for the “specificity” bias.

Second, it was possible to pool data for each included diagnostic categories only for sCD14 and antibodies again bacterial endotoxin. So, trans-diagnostic considerations extended to other biomarkers need to be further explored by future studies. Third, none of the studies provided direct measures of “severity of sickness behaviors.” Studies investigating each diagnostic category used a different severity scale. This is particularly relevant for studies on schizophrenia, where only negative, but not depressive, symptoms were measured. However, as highlighted in Table 1, it is clear how symptoms definitions overlap across different scales and diagnoses.

Fourth, new evidence questioned the reliability of zonulin as a biomarker of intestinal permeability because of inadequacy of commercially available enzyme assays. However, our results rely on multiple biomarkers of gut dysbiosis, reducing the possibility of biased interpretations.

Finally, the pooled estimate for A-1-AT and sCD14 was characterised by high heterogeneity, even when accounted for by sensitivity analyses. These results warrant exploration in larger, longitudinal cohorts.

## Conclusions

Our findings suggest that gut dysbiosis may underlie symptoms of sickness behavior across traditional diagnostic boundaries and provide evidence for future investigations on a trans-diagnostic target in an area of unmet clinical need.

## Supplementary information

Supplemental material
