# Supplementary material for: Gut dysbiosis in severe mental illness and chronic fatigue: a novel trans-diagnostic construct? A systematic review and meta-analysis
Source: Mol Psychiatry. 2021 Feb 8;27(1):141–53. doi: 10.1038/s41380-021-01032-1 (PMC8960409; doi:10.1038/s41380-021-01032-1)
Supplement: Supplementary file 1 — Supplemental material [file 41380_2021_1032_MOESM1_ESM.docx]

**Supplementary Online Content**

[Moose Checklist 2](#_Toc59181958)

[PROSPERO Research Protocol 4](#_Toc59181959)

[eFigure1. PRISMA Flow Chart 8](#_Toc59181960)

[eResults. Influence of medication on gut-dysbiosis biomarker levels 9](#_Toc59181961)

[eFigure2. Meta-analysis of gut-dysbiosis biomarkers in medicated vs. un-medicated patients 10](#_Toc59181962)

[Impact of BMI and smoking status on main findings 10](#_Toc59181963)

[eTable. Included studies investigating bipolar disorder – analytic considerations based on illness phase 11](#_Toc59181964)

[Excluded Studies and Reasons for Exclusion 12](#_Toc59181965)

| **Item No** | **Moose Checklist for Observational Studies [1]**  **Recommendation** | **Reported on Page No** |
| --- | --- | --- |
| Reporting of background should include | | |
| 1 | Problem definition | 2 |
| 2 | Hypothesis statement | 3 |
| 3 | Description of study outcome(s) | 4 |
| 4 | Type of exposure or intervention used | 3-4 |
| 5 | Type of study designs used | 4 |
| 6 | Study population | 4 |
| Reporting of search strategy should include | | |
| 7 | Qualifications of searchers (eg, librarians and investigators) | 4, Title page |
| 8 | Search strategy, including time period included in the synthesis and key words | 3-4 |
| 9 | Effort to include all available studies, including contact with authors | 4 |
| 10 | Databases and registries searched | 4 |
| 11 | Search software used, name and version, including special features used (eg, explosion) | 4 |
| 12 | Use of hand searching (eg, reference lists of obtained articles) | 4 |
| 13 | List of citations located and those excluded, including justification | Supp. eTable1 |
| 14 | Method of addressing articles published in languages other than English | Supp. eMethods |
| 15 | Method of handling abstracts and unpublished studies | Supp. eMethods |
| 16 | Description of any contact with authors | - |
| Reporting of methods should include | | |
| 17 | Description of relevance or appropriateness of studies assembled for assessing the hypothesis to be tested | 4 |
| 18 | Rationale for the selection and coding of data (eg, sound clinical principles or convenience) | 4 |
| 19 | Documentation of how data were classified and coded (eg, multiple raters, blinding and interrater reliability) | 4 |
| 20 | Assessment of confounding (eg, comparability of cases and controls in studies where appropriate) | 4 |
| 21 | Assessment of study quality, including blinding of quality assessors, stratification or regression on possible predictors of study results | 4 |
| 22 | Assessment of heterogeneity | 4 |
| 23 | Description of statistical methods (eg, complete description of fixed or random effects models, justification of whether the chosen models account for predictors of study results, dose-response models, or cumulative meta-analysis) in sufficient detail to be replicated | 4 |
| 24 | Provision of appropriate tables and graphics | Table 2, Table 3, Figure 2 |
| Reporting of results should include | | |
| 25 | Graphic summarizing individual study estimates and overall estimate | Figure 2 |
| 26 | Table giving descriptive information for each study included | Table 2 |
| 27 | Results of sensitivity testing (eg, subgroup analysis) | 8 |
| 28 | Indication of statistical uncertainty of findings | 8 |

| **Item No** | **Recommendation** | **Reported on Page No** |
| --- | --- | --- |
| Reporting of discussion should include | | |
| 29 | Quantitative assessment of bias (eg, publication bias) | Figure 2 |
| 30 | Justification for exclusion (eg, exclusion of non-English language citations) | Supp. eTable 1 |
| 31 | Assessment of quality of included studies | 7-8 |
| Reporting of conclusions should include | | |
| 32 | Consideration of alternative explanations for observed results | 8 |
| 33 | Generalization of the conclusions (ie, appropriate for the data presented and within the domain of the literature review) | 9 |
| 34 | Guidelines for future research | 8 |
| 35 | Disclosure of funding source | 9 |

**PROSPERO Research Protocol (CRD42019135329)**

**[Note:** The original protocol planned to investigate biomarkers of gut dysbiosis in psychiatric disorders, as well as in autism spectrum disorder and alcoholism. However, we decided to focus the scope of the paper on the disorders with shared sickness behavior symptoms**]**

**PROSPERO Registered Protocol**

**Review Question**

Recent findings on the gut-microbiome and gut-brain axis have suggested an association between intestinal permeability and psychiatric disorders. Increased gut permeability and the resulting translocation of bacterial markers from the gut lumen to the bloodstream may enhance a systemic pro-inflammatory status, which is believed to play a key role across many psychiatric disorders. Blood biomarkers for bacterial translocation and increased gut permeability may therefore provide a novel tool for diagnostic and prognostic use in psychiatric disorders.
This study aims to explore the clinical relevance of intestinal permeability biomarkers across various psychiatric disorders, such as schizophrenia, bipolar disorder, major depressive disorder, autism spectrum disorder, chronic fatigue syndrome, and alcohol dependence.

**Searches**

The search will be conducted from inception to July 2019 in Web of Science and PubMed. Articles not in English will be excluded.

Search terms:

“Depression” OR “depressed” OR “MDD” OR “major depressive disorder” OR “dysthymia” OR “schizophrenia” OR “schizoaffective disorder” OR “psychotic disorder” OR “psychosis” OR “delusion” OR “bipolar disorder” OR “bipolar” OR “BPD” OR “BD” OR “mania” OR “hypomania” OR “autism” OR “autism spectrum disorder” OR “ASD” OR “chronic fatigue syndrome” OR “chronic fatigue” OR “CFS” OR “alcoholism” OR “alcohol dependence” OR “alcohol abuse”

AND

LPS OR lipopolysaccharide OR “gram-negative bacteria” OR LBP OR “liposaccharide binding protein” OR sCD14 OR “soluble CD14” OR “I-FABP” OR “intestinal fatty acid binding protein” OR Cr-EDTA OR “zonulin” OR “tight junction protein” OR CLDN-1 OR OCLN OR TRIC OR “ASCA” OR “IgG Saccharomyces cerevisiae” OR “IgA Saccharomyces cerevisiae” OR “IgM Saccharomyces cerevisiae” OR “IgG gram-negative bacteria” OR “IgA gram-negative bacteria” OR OR “IgM gram-negative bacteria” OR “IgG LPS” OR “IgA LPS” OR “IgM LPS” “endotoxins” OR “LPS" OR “calprotectin" OR "alpha-1-antitrypsin” OR “A-1-AT”

Please note that search terms regarding psychiatric syndromes and intestinal permeability biomarkers were based on a preliminary search of the literature and after consultation with an expert in the field (P.B.)

**Types of studies to be included**

Inclusion criteria:

- Case control studies reporting data on easily-accessible biomarkers (e.g., plasma) for intestinal permeability and bacterial translocation. Studies need to include participants with a diagnosis of at least one of the following, according to internationally validated criteria: major depressive disorder, schizophrenia, bipolar disorder, autism spectrum disorder, chronic fatigue syndrome, alcohol dependence, alcohol abuse

We will include studies reporting data on in- or out-patients at any stage of illness, independent of their medication status.

- Studies investigating the relationship between easily-accessible biomarkers (e.g., blood) for intestinal permeability and bacterial translocation, symptoms severity and treatment response in individuals with a diagnosis of: major depressive disorder, schizophrenia, bipolar disorder, autism spectrum disorder, chronic fatigue syndrome, alcohol dependence, alcohol abuse
- Studies published in English.

Exclusion criteria:

- Reviews, studies published in conference abstracts, letters to the editor

**Condition or domain being studied**

1. Major Depressive Disorder (MDD). We will include studies reporting data for patients diagnosed with MDD.
2. Schizophrenia. We will include studies reporting data for patients diagnosed with schizophrenia, at any stage of illness.
3. Bipolar Disorder. We will include studies reporting data for patients diagnosed with bipolar disorder.
4. Autism Spectrum Disorder (ASD). We will include studies reporting data for patients diagnosed with ASD.
5. Chronic Fatigue Syndrome (CFS). We will include studies reporting data for patients diagnosed with CFS.
6. Alcohol Dependence. We will include studies reporting data for patients diagnosed with alcohol dependence.
7. We will be selecting studies that compare data for intestinal permeability biomarkers between patients diagnosed with one of the disorders above (1-6) and healthy controls.

The list of “conditions being studied” was based on a preliminary search of the scientific literature investigating gut-permeability biomarkers across psychiatric disorders.

**Participants/population**

Participants will have a diagnosis of one or more of the following according to internationally validated criteria: major depressive disorder, schizophrenia, bipolar disorder, autism spectrum disorder, chronic fatigue syndrome, alcohol dependence, alcohol abuse.

**Intervention(s), exposure(s)**

Levels of easily-accessible biomarkers (e.g. blood) for intestinal permeability and/or bacterial translocation in individuals with a diagnosis of one of the following: major depressive disorder, schizophrenia, bipolar disorder, autism spectrum disorder, chronic fatigue syndrome, alcohol dependence, alcohol abuse.

**Comparator(s)/control**

Levels of easily-accessible biomarkers (e.g. blood) for intestinal permeability and/or bacterial translocation in healthy controls.

**Main outcome(s)**

We will investigate the clinical relevance of intestinal permeability and/or bacterial translocation markers across psychiatric disorders, by:

(1) comparing the differences in these markers between patients and healthy controls (primary outcome);

(2) correlation analysis between severity of symptoms and these markers in patients;

(3) Illness stage-related modifications of these markers in patients, compared with healthy controls

(4) Association between the modifications of these markers and the response to treatment (secondary outcomes).

**Data extraction (selection and coding)**

Following an initial literature search as described above, two authors (JMS and AR) will examine study titles and abstracts to assess eligibility for inclusion, where there is doubt about eligibility the full text of the article will be reviewed and if doubt remains there will be discussion between researchers. Where there are disagreements between the two authors responsible for initial abstract screening, AM will arbitrate. Data will be extracted on the patient group and control group. We will extract data on the type of biomarkers analyzed, the type of bodily fluid from where these markers were obtained (e.g., whole blood, plasma, urine), magnitude of difference between patients and controls, statistical significance of any between group difference, any statistically significant correlation biomarkers for intestinal permeability and symptoms in patients. We will also extract data relevant for interpretation of results, such as number of participants, gender, stage, phase, medication status and type of medication received, and duration of illness.

**Risk of bias (quality) assessment**

Risk of bias will be assessed for individual papers based on an adapted form of the Newcastle-Ottawa scale, assessing selection of participants, comparability of study groups, and methods of data collection for key outcomes and confounders.

**Strategy for data synthesis**

We will conduct a qualitative synthesis of results of all relevant studies that meet the inclusion criteria. If feasible, individual meta-analyses investigating differences between patients and healthy controls (aggregate data) will be performed for each gut-biomarker (with two or more available studies) for all psychiatric syndromes combined and then for each syndrome separately.

Data will be synthetized in order to obtain summary standardised mean differences (SMDs) in peripheral concentrations of gut-permeability biomarkers between patients and healthy controls.

Between-study heterogeneity will be assessed by calculating Higgins I^2^ based on Cochrane’s Q

Because we expect a moderate to high heterogeneity across studies, a random-effect model will be used. Statistical analysis will be conducted in STATA.

**Analysis of subgroups or subsets**

None**.**

# **eFigure1. PRISMA Flow Chart**

**
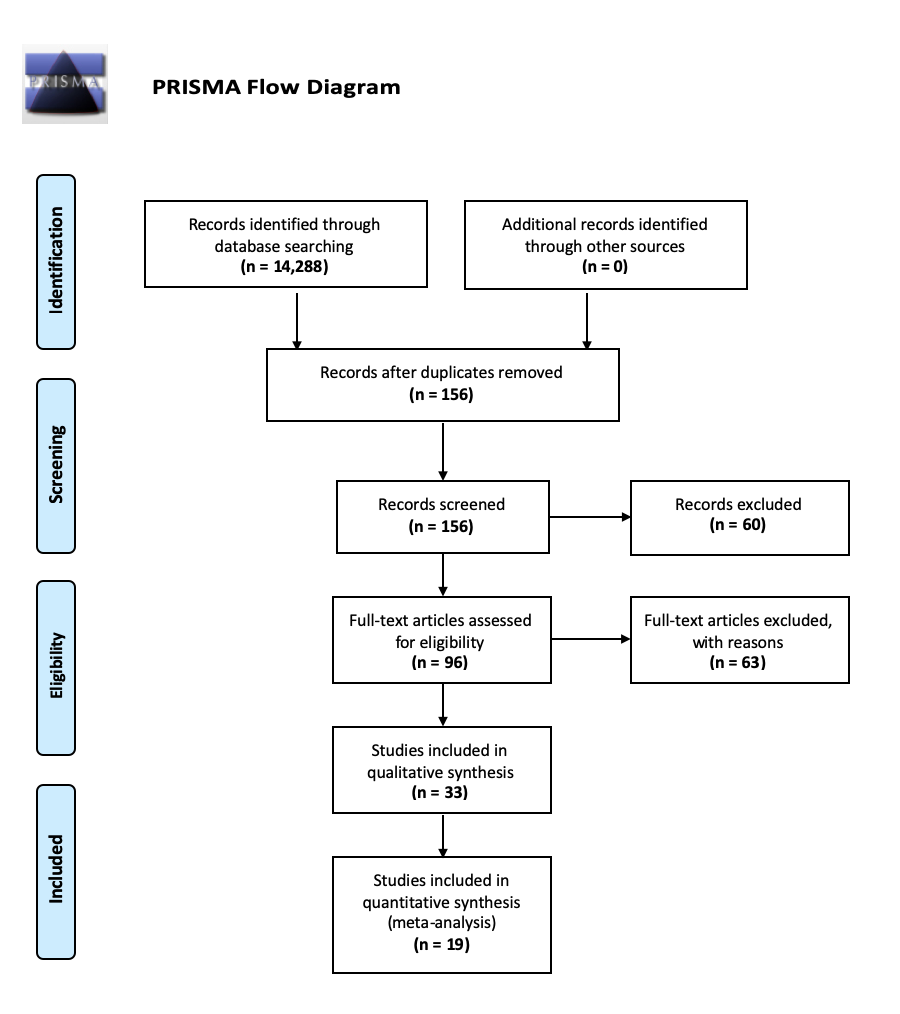
**

#

# **eResults. Influence of medication on gut-dysbiosis biomarker levels**

To control for the possibility of medication as a confounding factor, we investigated if biomarker levels differed between medicated (N=200) and un-medicated (N=99) patients (eFigure1). Four studies provided data for this meta-analysis; one study reported IgG to endobacteria [2], and three studies were on alpha-1-antitrypsin [3–5]. The pooled estimate shows that intestinal inflammation and bacterial translocation did not differ significantly between medicated and un-medicated patients (SMD =-0.68 ;95% Cl = -1.88 to 0.52; *P =* 0.26; *I^2^* = 93.89%). Subgroup analysis shows this is consistent across the psychiatric disorders. Removing one study [2] so the analysis is only on A-1-AT levels accounts for the high heterogeneity but does not alter the outcome of the analysis (SMD = -0.14; 95% Cl = -0.48 - 0.21; *p =* 0.44; *I^2^* = 0%).

Nineteen [2, 4–21] of the thirty-two studies included in the qualitative analysis, included patients on medication. This consisted of twelve of the studies investigating SCZ [2, 4–8, 10, 11, 14–16, 19], nine studies on BPD [4, 9, 11–13, 15, 17, 20, 21], and six on MDD patients [4, 9, 15, 18, 21]. None of the CFS studies included information on medication as a confounding factor. All nineteen studies report that medication is not responsible for elevation of biomarkers of gut-dysbiosis in patients with psychiatric illness. Only one study reports a significant difference, reporting significantly higher circulating levels of IgG antibodies to Saccharomyces cerevisiae (ASCA) in anti-psychotic naïve SCZ patients when compared to medicated patients [2].

One study [20] explored the effect of medication on loosening of epithelial tight junctions in BPD patients, and showed no relationship between levels of circulating immunoglobulin against tight junction proteins, Zonulin and Claudin-5, and medication type.

Three studies, two on SCZ [19, 22] and one on BPD [13], report no significant difference in circulating levels of sCD14 between medicated and unmedicated patients. One of these studies also reported no significant difference in levels of LBP in medicated SCZ patients [22]. Seven studies investigated if there is an association with medication type on the circulating levels of antibodies to endotoxins [2, 6–8, 12, 14, 21]. This included five studies on SCZ [2, 6–8, 14]

(three of which were from the same research group), three studies were in BPD patients [9, 12, 21] and one study on MDD [21]. Six of these studies showed that no significant effect of drug type on circulating immunoglobin response to endotoxins was consistent across SCZ [6–8, 14], BPD [23–25] and MDD [25] patients. One study on SCZ patients [2], reports significantly higher circulating levels of ASCA IgG in anti-psychotic naïve patients when compared to medicated patients.

Two studies, both on SCZ patients, explored the influence of medication on intestinal inflammation, and report that circulating levels of alpha-1-antitrypsin was unaffected by medication status [4] or drug type [5].

# **eFigure2. Meta-analysis of gut-dysbiosis biomarkers in medicated vs. un-medicated patients**

| 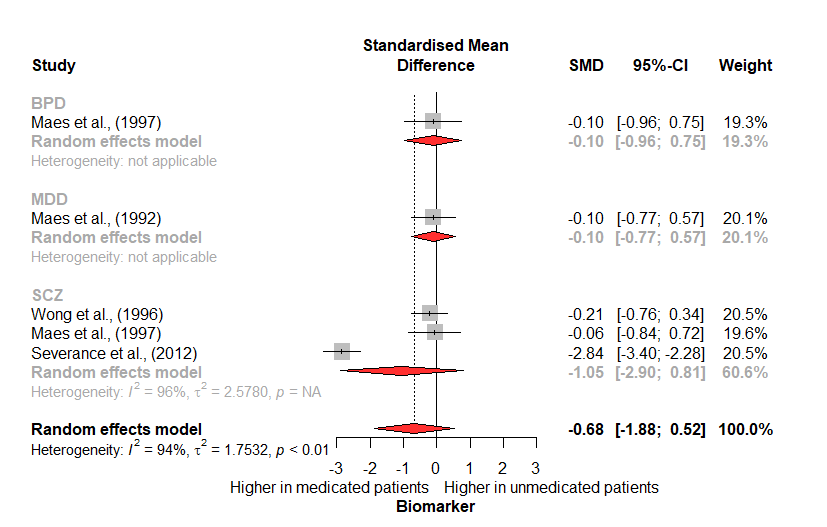 |
| --- |

*Meta-analysis of circulating biomarker levels in medicated patients compared to unmedicated patients. Severance et al. (2012) provided data on antibodies to bacterial endotoxins, the other studies report levels of circulating alpha-1-antitryspin.*

# **Impact of BMI and smoking status on main findings**

| The included studies either: 1) Had no significant difference in BMI and smoking status between patients and controls. References in main text: [20, 26, 31, 37, 39] 2) Adjusted their analysis for differences in BMI and smoking status between subject groups  References in main text: [9, 12, 23, 24, 28, 29, 30, 33, 35, 38, 40, 41, 46] 3) Did not mention or report an effect of BMI and smoking on results  References in main text: [13, 19, 21, 22, 25, 27, 32, 36, 42, 43, 44, 45, 49, 51] |
| --- |

# **eTable. Included studies investigating bipolar disorder – analytic considerations based on illness phase**

| **Study** | **Biomarker** | **Phase of Bipolar Disorder** | **Main findings** | **Considerations for meta-analytic estimates** |
| --- | --- | --- | --- | --- |
| *Maes et al., 1997*  Ref. 20 in the main text | A-1-AT  (Blood) | Mania | = A-1-AT  In patients vs controls | Excluded in sensitivity analysis |
| *Severance et al., 2013*  Ref. 22 in the main text | sCD14; LBP  (Blood) | Not specified | ↑ sCD14 in patients vs controls  =LBP in patients vs controls | Included in the analysis |
| *Severance et al., 2014*  Ref. 32 in the main text | ASCA IgG  (Blood) | Mixed sample (mania, depression, and mixed episode) | ↑ ASCA IgG in patients v. controls | Excluded in sensitivity analysis |
| *Jakobsson et al., 2015*  Ref. 9 in the main text | sCD14  (Blood) | Euthymic | ↑ sCD14 in patients vs controls | Included in the analysis |
| *Tanaka T et al.,*  *2017*  Ref. 13 in the main text | sCD14  (Blood) | Not specified, outpatients | ↑ sCD14 in patients vs controls | Included in the analysis |
| *Kılıç F et al., 2020*  Ref. 33 in the main text | Zonulin  (Blood) | Mixed (euthymic and manic) | ↑ Zonulin in euthymic patients vs controls | As data were available from both euthymic and manic patients, we re-ran the analysis excluding data from manic patients. |

# **Excluded Studies and Reasons for Exclusion**

| **Reference** | **Reason(s) for Exclusion** |
| --- | --- |
| Leclercq et al. (2014) | LPS-stimulated response in PBMCs |
| Bala et al. (2014) | Healthy subjects only |
| Harada et al. (1985) | Diagnostic criteria unclear |
| Mutlu et al. (2012) | No biomarkers of interest reported |
| Kliushnik et al. (2011) | Non-English publication |
| Rose et al. (2018) | No biomarkers of interest reported |
| Jyonouchi et al. (2002) | LPS-stimulated response in PBMCs |
| Nadeem et al. (2016) | No biomarkers of interest reported |
| Kozłowska et al. (2017a) | No biomarkers of interest reported |
| Maes et al. (2008) | No biomarkers of interest reported |
| Hung et al. (2014) | No biomarkers of interest reported |
| Torres et al. (2013) | No biomarkers of interest reported |
| Kozłowska et al. (2017b) | No biomarkers of interest reported |
| Kiecolt-Glaser et al. (2018) | No formal diagnosis of subjects |
| Eutneuer et al. (2017) | No biomarkers of interest reported |
| Roomruangwong et al. (2017) | Pregnant subjects |
| Kéri et al. (2014) | No biomarkers of interest reported |
| Szczepocka et al. (2017) | No biomarkers of interest reported |
| Rozing et al. (2018) | No biomarkers of interest reported |
| Subbotskaya et al. (2015) | Non-English publication |
| Lyons et al. (2016) | Letter to the editor |
| Yin Yee et al. (2017) | No serum biomarkers reported, only gene expression |
| Greene et al. (2017) | Mice model |
| Kliushnik et al. (2014) | Non-English publication |
| Kliushnik et al. (2009) | Non-English publication |
| Farrell et al. (2019) | Case report |
| Reale et al. (2011) | No biomarkers of interest reported |
| Zozulia et al. (2011) | Non-English publication |
| Ignlot et al. (1994) | LPS-stimulated leukocytes |
| Parlesak et al. (2000) | No formal diagnosis |
| Zhou et al. (2018) | Pregnant subjects |
| Rudduck et al. (1985) | No serum biomarkers reported, only gene expression |
| Fiorentino et al. (2016) | Post-mortem samples |
| Zhang et al. (2020) | Genome study |
| Monnig et al. (2019) | No formal diagnosis |
| Maes et al. (2015) | Stimulated leukocytes |
| Maes et al. (2012) | Review article |
| Özyurt et al. (2018) | No diagnosis of interest |
| Gu et al. (2020) | Mice model |
| Lisi et al. (2013) | Measured levels in stimulated PBMCs |
| Bajaj et al. (2019) | No diagnosis of interest |
| Meinitzer et al. (2020) | No formal diagnosis |
| Deng et al. (2019) | No formal diagnosis |
| Pietrukaniec et al. (2019) | No formal diagnosis |
| Chan et al. (2017) | No biomarkers of interest reported |
| Ramsey et al. (2013) | No biomarkers of interest reported |
| Maes et al. (2015) | No data for biomarkers of interest reported |
| Esnafoglu et al. (2017) | No longer diagnosis of interest (ASD) |
| Józefczuk, et al. (2018) | No longer diagnosis of interest (ASD) |
| Babinská et al. (2017) | No longer diagnosis of interest (ASD) |
| Sarsella et al. (2016) | No longer diagnosis of interest (ASD) |
| Cortelazzo et al. (2016) | No longer diagnosis of interest (ASD) |
| Iovene et al. (2017) | No longer diagnosis of interest (ASD) |
| Magistris et al. (2010) | No longer diagnosis of interest (ASD) |
| Emanuele et al. (2010) | No longer diagnosis of interest (ASD) |
| Pusponegoro et al. (2015) | No longer diagnosis of interest (ASD) |
| Carissimi et al. (2019) | No longer diagnosis of interest (ASD) |
| Liangpunsakul et al. (2017) | No longer diagnosis of interest (Alcohol Dependency) |
| Joyce et al. (1992) | No longer diagnosis of interest (Alcohol Dependency) |
| Addolorato et al. (2020) | No longer diagnosis of interest (Alcohol Dependency) |
| Leclercq et al. (2012) | No longer diagnosis of interest (Alcohol Dependency) |

**eReferences**

1. Stroup DF. Meta-analysis of Observational Studies in EpidemiologyA Proposal for Reporting. JAMA. 2000;283:2008.

2. Severance EG, Alaedini A, Yang S, Halling M, Gressitt KL, Stallings CR, et al. Gastrointestinal inflammation and associated immune activation in schizophrenia. Schizophr Res. 2012;138:48–53.

3. Maes M. Higher α1-antitrypsin, haptoglobin, ceruloplasmin and lower retinol binding protein plasma levels during depression: Further evidence for the existence of an inflammatory response during that illness. J Affect Disord. 1992;24:183–192.

4. Maes M, Delange J, Ranjan R, Meltzer HY, Desnyder R, Cooremans W, et al. Acute phase proteins in schizophrenia, mania and major depression: modulation by psychotropic drugs. Psychiatry Res. 1997;66:1–11.

5. Wong CT, Tsoi WF, Saha N. Acute phase proteins in male Chinese schizophrenic patients in Singapore. Schizophr Res. 1996;22:165–171.

6. Maes M, Sirivichayakul S, Kanchanatawan B, Vodjani A. Upregulation of the Intestinal Paracellular Pathway with Breakdown of Tight and Adherens Junctions in Deficit Schizophrenia. Mol Neurobiol. 2019;56:7056–7073.

7. Maes M, Kanchanatawan B, Sirivichayakul S, Carvalho AF. In Schizophrenia, Increased Plasma IgM/IgA Responses to Gut Commensal Bacteria Are Associated with Negative Symptoms, Neurocognitive Impairments, and the Deficit Phenotype. Neurotox Res. 2019;35:684–698.

8. Maes M, Sirivichayakul S, Kanchanatawan B, Vodjani A. Breakdown of the Paracellular Tight and Adherens Junctions in the Gut and Blood Brain Barrier and Damage to the Vascular Barrier in Patients with Deficit Schizophrenia. Neurotox Res. 2019;36:306–322.

9. Maes M, Simeonova D, Stoyanov D, Leunis J-C. Upregulation of the nitrosylome in bipolar disorder type 1 (BP1) and major depression, but not BP2: Increased IgM antibodies to nitrosylated conjugates are associated with indicants of leaky gut. Nitric Oxide Biol Chem. 2019;91:67–76.

10. Yang Y, Wan C, Li H, Zhu H, La Y, Xi Z, et al. Altered Levels of Acute Phase Proteins in the Plasma of Patients with Schizophrenia. Anal Chem. 2006;78:3571–3576.

11. Severance EG, Gressitt KL, Stallings CR, Origoni AE, Khushalani S, Leweke FM, et al. Discordant patterns of bacterial translocation markers and implications for innate immune imbalances in schizophrenia. Schizophr Res. 2013;148:130–137.

12. Severance EG, Gressitt KL, Yang S, Stallings CR, Origoni AE, Vaughan C, et al. Seroreactive marker for inflammatory bowel disease and associations with antibodies to dietary proteins in bipolar disorder. Bipolar Disord. 2014;16:230–240.

13. Jakobsson J, Bjerke M, Sahebi S, Isgren A, Johan Ekman C, Sellgren C, et al. Monocyte and microglial activation in patients with mood-stabilized bipolar disorder. J Psychiatry Neurosci. 2015;40:250–258.

14. Dickerson F, Stallings C, Origoni A, Schroeder J, Katsafanas E, Schweinfurth L, et al. Inflammatory Markers in Recent Onset Psychosis and Chronic Schizophrenia. Schizophr Bull. 2016;42:134–141.

15. Dickerson F, Severance E, Yolken R. The microbiome, immunity, and schizophrenia and bipolar disorder. Brain Behav Immun. 2017;62:46–52.

16. Chan MK, Cooper JD, Heilmann-Heimbach S, Frank J, Witt SH, Nöthen MM, et al. Associations between SNPs and immune-related circulating proteins in schizophrenia. Sci Rep. 2017;7:12586.

17. Tanaka T, Matsuda T, Hayes LN, Yang S, Rodriguez K, Severance EG, et al. Infection and inflammation in schizophrenia and bipolar disorder. Neurosci Res. 2017;115:59–63.

18. Alvarez-Mon MA, Gómez AM, Orozco A, Lahera G, Sosa MD, Diaz D, et al. Abnormal Distribution and Function of Circulating Monocytes and Enhanced Bacterial Translocation in Major Depressive Disorder. Front Psychiatry. 2019;10.

19. Mørch RH, Dieset I, Færden A, Reponen EJ, Hope S, Hoseth EZ, et al. Inflammatory markers are altered in severe mental disorders independent of comorbid cardiometabolic disease risk factors. Psychol Med. 2019;49:1749–1757.

20. Kılıç F, Işık Ü, Demirdaş A, Doğuç DK, Bozkurt M. Serum zonulin and claudin-5 levels in patients with bipolar disorder. J Affect Disord. 2020;266:37–42.

21. Simeonova D, Stoyanov D, Leunis J, Carvalho AF, Kubera M, Murdjeva M, et al. Increased Serum Immunoglobulin Responses to Gut Commensal Gram-Negative Bacteria in Unipolar Major Depression and Bipolar Disorder Type 1, Especially When Melancholia Is Present. Neurotox Res. 2020;37:338–348.

22. Severance EG, Gressitt KL, Stallings CR, Origoni AE, Khushalani S, Leweke FM, et al. Discordant patterns of bacterial translocation markers and implications for innate immune imbalances in schizophrenia. Schizophr Res. 2013;148:130–137.

23. Maes M, Simeonova D, Stoyanov D, Leunis J. Upregulation of the nitrosylome in bipolar disorder type 1 (BP1) and major depression, but not BP2: Increased IgM antibodies to nitrosylated conjugates are associated with indicants of leaky gut. Nitric Oxide. 2019;91:67–76.

24. Simeonova D, Stoyanov D, Leunis J, Carvalho AF, Kubera M, Murdjeva M, et al. Increased Serum Immunoglobulin Responses to Gut Commensal Gram-Negative Bacteria in Unipolar Major Depression and Bipolar Disorder Type 1, Especially When Melancholia is Present. MEDICINE & PHARMACOLOGY; 2019.

25. Severance EG, Gressitt KL, Yang S, Stallings CR, Origoni AE, Vaughan C, et al. Seroreactive marker for inflammatory bowel disease and associations with antibodies to dietary proteins in bipolar disorder. Bipolar Disord. 2014;16:230–240.
